# Medication overuse headache in Europe and Latin America: general demographic and clinical characteristics, referral pathways and national distribution of painkillers in a descriptive, multinational, multicenter study

**DOI:** 10.1186/s10194-016-0612-2

**Published:** 2016-03-08

**Authors:** Ninett Louise Find, Rossana Terlizzi, Signe Bruun Munksgaard, Lars Bendtsen, Cristina Tassorelli, Giuseppe Nappi, Zaza Katsarava, Miguel Lainez, Maria Teresa Goicochea, Beatriz Shand, Ricardo Fadic, Santiago Spadafora, Marco Pagani, Rigmor Jensen, COMOESTAS Consortium

**Affiliations:** Faculty of Health Science, University of Copenhagen, Copenhagen, Denmark; Danish Headache Center, Neurological Department, Glostrup Hospital, Glostrup, Denmark; Department of Biomedical and NeuroMotor Sciences (DIBINEM), Alma Mater Studiorum, Bologna University, Ospedale Bellaria, Bologna, Italy; Headache Science Center, C. Mondino National Neurological Institute C. Mondino Foundation, Dept. of Brain and Behavioral Sciences, University of Pavia, Pavia, Italy; Department of Neurology, University Hospital of Essen, Essen, Germany; Foundation of the Valencian Community, University Clinical Hospital, Barcelona, Spain; Foundation for Combating Neurological Diseases of Childhood, Buenos Aires, Argentina; Pontificia Universidad Católica de Chile, Santiago, Chile; Universidad Isalud, Buenos Aires, Argentina; Bioengineering and Medical Informatics Consortium, Pavia, Italy

**Keywords:** Medication overuse headache, Healthcare utilization, Overuse of acute medication, International variation

## Abstract

**Background:**

Medication overuse headache (MOH) is a very disabling and costly disorder due to indirect costs, medication and healthcare utilization. The aim of the study was to describe general demographic and clinical characteristics of MOH, along with the national referral pathways and national painkillers distribution in several European and Latin American (LA) Countries.

**Methods:**

This descriptive cross-sectional observational study included 669 patients with MOH referred to headache-centers in Europe and LA as a part of the COMOESTAS project. Information about acute medication and healthcare utilization were collected by extensive questionnaires, supplemented with structured patient interviews.

**Results:**

Triptans were overused by 31 % European patients and by 6 % in LA (*p* < 0.001), whereas ergotamines were overused by 4 % in Europe and 72 % in LA (*p* < 0.001). Simple analgesics were overused by 54 % in Europe and by 33 % in LA (*p* < 0.001), while combination-analgesics were more equally overused (24 % in Europe and 29 % in LA). More European patients (57 %) compared with LA patients (27 %) visited general practitioners (*p* < 0.001), and 83 % of European patients compared to 38 % in LA consulted headache specialists (*p* < 0.001). A total of 20 % in Europe and 30 % in LA visited emergency rooms (*p* = 0.007).

**Conclusion:**

There are marked variations between LA and Europe in healthcare pathways and in acute medication overuse regarding patients with MOH. This should be considered when planning prevention campaigns against MOH.

**Electronic supplementary material:**

The online version of this article (doi:10.1186/s10194-016-0612-2) contains supplementary material, which is available to authorized users.

## Background

Medication overuse headache (MOH) is a globally prevalent and disabling chronic disorder, affecting up to 2 % of populations [1–6]. In Europe, MOH is a fast growing economic burden for society, due to reduced productivity at work, absenteeism from work, cost of medication, and healthcare-resource utilization [5].

Epidemiological studies performed on general populations in western countries report a high degree of healthcare use among patients suffering from chronic headache, often complicated by MOH, especially in the primary sector [2, 6, 7]. In addition, a smaller French study found a high number of contacts to the emergency room (ER) among patients with chronic headache with and without medication overuse [8].

However, healthcare systems differ considerably among nations in regards to organization, referral pathways and financing of healthcare costs. This may contribute to international variations in healthcare utilization among patients with MOH.

Similarly, cultural, economic and political differences may contribute to an international variation in the use of acute medications [3, 6, 7, 9, 10]. A Spanish epidemiological study reported simple analgesics as the most consumed type of drug, followed by ergotamines, among patients with chronic daily headache (CDH) and acute medication overuse [3]. Scandinavian studies report the same tendency towards a high preference for simple analgesics, accompanied by combination-analgesics [7, 10]. It has been described that patients with MOH in USA have a higher use of opioids and barbiturates compared with other nations [6, 9].

The literature regarding healthcare utilization and pattern of medication overuse among patients with MOH in Latin America (LA) is sparse, which also has been mentioned by Allena et al. [11]. A better characterization of this population may assist and direct prevention campaigns and relevant therapy of MOH. Recently, a paper of the COMOESTAS project concerning clinical and demographical characteristics of LA patients with MOH has been published [12].

The current study characterizes and compares the general demographic and clinical characteristics of MOH, the referral pathways and the pattern of acute medication distribution and overuse between European and LA patients suffering from MOH referred to headache centers, in a multinational, multicenter setup.

## Methods

### Study design

The present study is a descriptive, cross-sectional, observational part of the COMOESTAS project. The main objective of the COMOESTAS project was to compare MOH-relapse rates six months after a structured, multidisciplinary detoxification program between MOH patients using a newly developed, electronic headache-diary system or a paper headache diary, and the main results are in progress for publication [13]. The highly positive effect of the treatment program on disability, depression and anxiety has recently been published [14].

### Study population

Patients referred to six national headache centers or clinics in Germany (University Hospital, Essen), Denmark (Danish Headache Center, Copenhagen), Italy (C. Mondino National Neurological Institute, Pavia), Spain (University Clinical Hospital, Valencia), Argentina (Foundation for Combating Neurological Diseases of Childhood, Buenos Aires) and Chile (Pontificia Universidad Católica de Chile, Santiago) were included consecutively from August 2008 to February 2009.

Patients were included if they were diagnosed with MOH according to the revised ICHD-II MOH-criteria [15], and capable of filling in paper and/or electronic diaries. The MOH diagnosis was based on 2 months previous history and at least 1 month with headache diary, in total 3 months overuse. Exclusion criteria were a current diagnosis of co-existent, significant and complicating medical or psychiatric illnesses; significant overuse of ‘pure’ opioids (patients overusing combination drugs containing opioids were allowed), benzodiazepines and barbiturates; overuse of alcohol and other drugs of addiction; current treatment with migraine-prophylactic drugs, inefficacy of previous, adequate detoxification programs; pregnancy or breastfeeding; or inability to reliably provide medical history.

At inclusion in the program, baseline characteristics were collected using extensive questionnaires supplemented with structured patient interviews. Information about primary headache diagnoses referred to the time before medication overuse and was based on the ICHD-II criteria. Both MOH and primary headache diagnosis were also revised retrospectively according to ICHD-III [16].

### Questionnaire of referral pathway, financing of healthcare costs, pathways and subsidization of acute medication

In order to obtain relevant background information on the organization of national healthcare systems, all headache centers were asked how patients were referred to a headache specialist, and if referral was needed for ER. In addition, we gathered information on how headache-related healthcare costs were covered.

Also, background information about medication subsidization and pathways (over-the-counter (OTC) drugs versus prescription-requiring analgesics) was obtained.

### Healthcare utilization and acute medication overuse

Data concerning healthcare utilization and acute medication overuse were collected by extensive questionnaires supplemented with structured interviews at the time of inclusion in the program. Firstly, we focused on the proportion of patients having healthcare consultations (General practitioner (GP), headache specialist consultations, and ER visits) and instrumental investigations (EEG, MR- and CT-scans and X-rays) in the year preceding therapy. Headache specialist consultations mainly included visits at neurologists and in a few cases neuro-surgeons.

Secondly, this study focused on acute medication overuse at the time of MOH-diagnosis. This was reported as proportion of patients with specific drug-overuse: Triptans, ergotamines, simple analgesics, opioids, combination-analgesics (analgesics combined with codeine, caffeine and/or antiemetics) or poly-overuse (combination of acute analgesics without overuse of a single drug-type). A patient was diagnosed with more than one MOH subtype if more than one type of drug was overused simultaneously. In addition, proportions of patients with specific drug-overuse were reported in groups separated according to primary headache diagnosis (migraine, tension type headache (TTH) or migraine plus TTH).

All results are shown for the total population, grouped by European and LA headache centers, and sub-grouped by national headache centers.

### Statistics

Statistical Package for Social Sciences (SPSS) version 20 was used for statistics. Data were either shown as proportions or as mean and SD in brackets. Chi-square Fischer’s exact tests were used for 2 × 2 tables and Pearson’s chi-square tests were used for 2 × 3 tables in order to compare proportions between Europe and LA, while Student’s independent t-tests were used to compare means. A value of *p* <0.050 was considered as significant. All *p*-values were two-tailed.

### Ethical issues

Local ethics committees from all headache centers approved the study (the local ethical committee at Essen University Hospital, local ethical committee at Pavia University, local ethical committee at Valencia University Clinical Hospital, Research Ethics Committee at the Medical School of Pontificia Universidad Católica de Chile and the Ethics Committee and Biomedical Research at FLENI in Argentina). In Denmark, such studies are exempted from the approval process, as the study did not foresee any new pharmacological treatment or interventions and was hence approved without application. All included patients gave informed consent before taking part.

## Results

### Study population

**Fig. 1:**
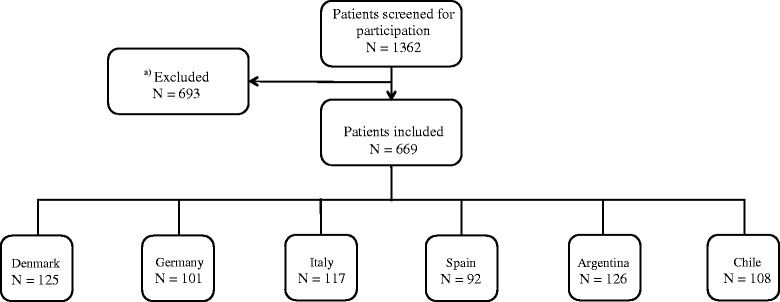
Study population. The flowchart illustrates the population. ^a)^Patients (*N* = 444) were excluded for 3 main reasons: previous detoxifications (mostly in the European Centers), wrong referral diagnosis (mostly in the LA Centers) and refusal to participate (equal distribution between EU and LA areas). Furthermore, 191 patients did not fullfill the criteria after filling out headache diary or did not have an internet spot available at home or nearby. Finally, 58 patients were excluded because they did not show up to the following visit or dataset was incomplete. Of the included patients, 435 came from Europe and 234 came from Latin America

A total of 1362 patients with potential MOH were screened in the 6 centers. Of these, 669 were enrolled in the study (Fig. 1).

The majority were 30–55 years old (67.7 %), females (79.4 %), and with onset of primary headache in their teens (64.4 %) (Table 1). The age, gender difference and age at onset of primary headache were very similar in all headache centers, and there was no significant difference between Europe and LA. Baseline characteristics including the proportions of primary headache diagnosis, marital status, educational level and headache frequency are shown in Table 1.

**Table 1:** Baseline Characteristics

		Denmark	Germany	Italy	Spain	Argentina	Chile	Europe	Latin America	*p*-value	Total
N		125	101	117	92	126	108	435	234		669
Age, years	<30	13.6	26.7	18.8	15.2	31.2	20.4	18.4	26.1		21.1
	30–55	71.2	63.4	72.6	70.7	58.4	70.4	69.7	63.7	0.058	67.7
	>55	15.2	9.9	8.5	14.1	10.4	9.3	12.0	9.8		11.2
Gender, female		77.6	74.3	82.1	80.4	80.2	81.5	78.6	80.8	0.55	79.4
High educational level^a^		87.2	40.4	71.8	49.5	80.2	67.6	64.4	74.4	0.009*	67.9
Marital status, married		70.4	52.0	58.1	68.1	50.8	55.6	62.4	53.0	0.021*	59.1
Primary headache diagnosis	Migraine^b^	30.4	80.2	82.9	46.7	72.2	79.6	59.5	75.6		65.2
	TTH^b^	26.4	2.0	0.9	16.3	5.6	1.9	11.7	3.8	<0.001*	9.0
	Migraine and TTH^b^	43.2	17.8	16.2	37.0	22.2	18.5	28.7	20.5		25.9
Age of onset of primary headache, years		23.4 (13.0)	18.1 (9.2)	14.1 (6.0)	18.9 (9.9)	16.2 (6.2)	19.7 (10.1)	18.8 (10.5)	17.8 (8.4)	0.22	18.4 (9.8)
Headache frequency, days/month^c^		25.8 (5.5)	25.1 (5.6)	23.3 (6.0)	23.6 (5.0)	22.1 (6.4)	23.0 (5.7)	24.5 (5.7)	22.5 (6.1)	<0.001*	23.8 (5.9)
Duration of overuse in years	<1	13.6	21.8	11.1	16.3	19.0	22.2	15.4	20.5		17.2
	1–5	67.2	54.5	58.1	57.6	61.9	57.4	59.8	59.8	0.13	59.8
	>5	19.2	22.4	30.8	26.1	19.0	20.4	24.8	19.7		23.0
EEG^d^		7.3	31.8	0.9	4.7	8.0	2.8	10.0	5.6	0.055	8.4
Scans (CT and MR)^d^		29.3	42.3	20.5	36.4	40.0	21.3	31.0	31.3	0.93	31.1
X-rays^d^		5.7	21.2	2.6	14.1	12.8	0.9	9.8	7.3	0.32	8.9

Age, gender, educational level, marital status, primary headache type: Proportion of patients in percent. Age of onset of primary headache and headache frequency: Mean (SD). Duration of overuse: Percent of patients categorized into three intervals. EEG, Scans and X-rays: Percent of patients, who had these instrumental investigations performed in the year preceding therapy

*TTH* tension type headache, *EEG* electro encephalogram, *CT* computer tomography scan, *MR* magnetic resonance scan

^a^High educational level = High/technical school or university degree

^b^Migraine = Migraine with aura, migraine without aura and chronic migraine. TTH = episodic and chronic forms

^c^Headache frequencies refer to the time of inclusion in the study and thereby to the MOH diagnosis

^d^ *N* = 643

*p < 0.05. p-values correspond to comparison between Europe and Latin America

Noteworthy, there were some significant differences in the proportion of primary headache diagnosis, although in general, most of patients had previous migraine or a combination of migraine and TTH, and only a minority had TTH as primary headache diagnosis (Table 1).

### Referral pathway and financing of healthcare costs

To be referred to a headache specialist in Denmark and Spain, patients need (Table 2) referral by their GP who function as gatekeeper. In Italy, patients need to be referred to a specialist by their GP, or can self-refer on private basis. In Germany, Argentina and Chile, patients could contact headache specialists directly by self-referral. There was free access for ER in all countries (Table 2).

**Table 2:** Referral pathway and financing of healthcare costs for patients

	Denmark	Germany	Italy	Spain	Argentina	Chile
Referral pathways						
Referral needed for headache specialist consultation	Yes	No	Yes or self-refer on private basis	Yes	No	No
Who are able to refer the patients to a headache specialist?	GP^a^	–	GP	GP	–	–
	Other specialists			Other specialists		
	ER^a^			ER		
	Other doctors					
Referral needed for emergency room visits	No	No	No	No	No	No
Financing of healthcare costs for patients						
Free Access	GP	GP	GP	GP	–	GP
	Headache Specialist	Headache Specialist	Headache Specialist^b^	Headache Specialist		–
	ER	ER	ER^c^	ER		ER
	EEG, MR-, CT-scan and X-rays	EEG, MR-, CT-scan and X-rays	EEG^b^, MR-^d^, CT-scan^d^ and X-rays^e^	EEG, MR-, CT-scan and X-rays		MR-, CT-scan and X-rays
Private healthcare assurance	–	–	–	–	GP	GP
					Headache Specialist	Headache Specialist
					ER	ER
					EEG, MR-, CT-scan and X-rays	EEG, MR-, CT-scan and X-rays
Out of own pocket	–	–	–	–	–	GP
			Headache Specialist^b^		Headache Specialist	Headache Specialist
			ER^c^		–	ER
			EEG^b^, MR-^d^, CT-scan^d^ and X-rays^d^		–	EEG, MR-, CT-scan and X-rays

*GP* general practitioner, *ER* emergency room, *EEG* electro encephalogram, *CT* computer tomography scan, *MR* magnetic resonance scan

^a^Referral after a relevant therapy course performed by a general neurologist

^b^Patients < 6 years or > 65 years are exempted for covering the healthcare costs. Without exemption contribution is required according to income

^c^Free access if appropriate request (real emergency)

^d^Patients < 6 years or > 65 years are exempted for covering the healthcare costs. Without exemption contribution is required depending on income

Free access = government covers the costs

In the European countries, the governments finance headache specialist consultations in full, except in Italy, where a partial contribution is requested from the patients. In LA, the costs of headache specialist consultations are covered by private healthcare assurances or out of own pocket. Financing of costs of instrumental investigations is presented in Table 2.

### Headache-related healthcare utilization

**Fig. 2:**
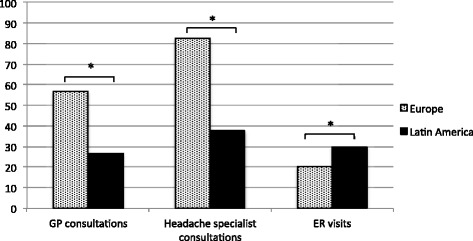
Headache-related healthcare consultations among patients with medication overuse headache in Europe and Latin America. The bar chart compares the proportion of patients (in percent) from Europe and Latin America with general practitioner (GP) consultations, headache specialist consultations and emergency room (ER) visits in the year preceding inclusion of the study. *p < 0.05

**Fig. 3:**
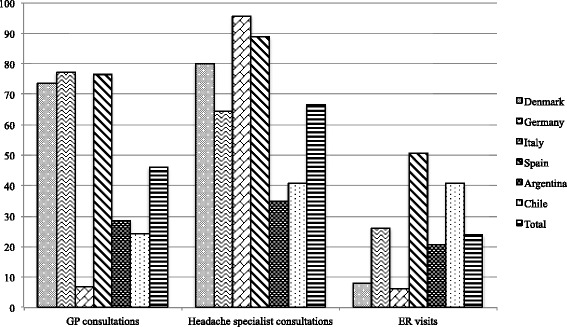
Healthcare consultations among patients with medication overuse headache. The bar chart presents the proportion of patients (in percent) from each headache clinic with general practitioner (GP) consultations, headache specialist consultations and emergency room (ER) visits in the year preceding inclusion of the study

Only 26.5 % of the LA patients had GP consultations for their headache in the year preceding inclusion in the study, compared with 56.7 % of the European patients (*p* < 0.001) (Fig. 2). It should be mentioned that only 6.8 % of the Italian patient group were screened through preliminary GP visits, which is far lower than the proportion of patients from LA (Fig. 3). In contrast, 77.1 % of the German patients had GP consultations, and of those, 35.4 % visited the GP more than 6 times per year, corresponding to a visit more than every second month (see Additional file 1: Table S1).

The vast majority of European patients (82.7 %), especially the Italian group (95.7 %), had seen a headache specialist prior to admission to the headache center, while this was only the case for 37.6 % of the LA patients (*p* < 0.001) (Figs. 2 and 3).

In LA, 29.9 % of the patients had headache-related ER visits, compared to 20.4 % of the European patients (*p* = 0.007) (Fig. 2). The proportions of patients with ER visits varied considerably among all centers, e.g., in Spain, 50.6 % of the patients visited the ER, in contrast to only 6.0 % of the Italian patient group and 20.6 % of the Argentinian patient group (Fig. 3).

The frequencies of headache-related instrumental investigations are presented in Table 1. There was no significant difference between Europe and LA.

### Characterization of medication overuse patterns

**Fig. 4:**
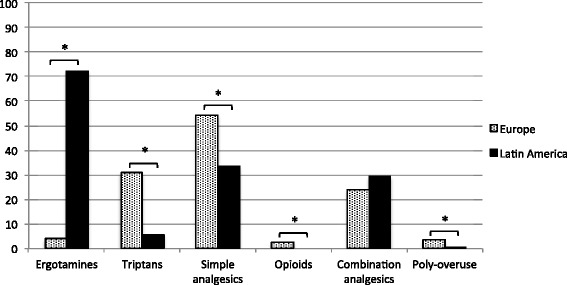
Type of overused analgesics in Europe and Latin America. The bar chart compares the proportion of patients (in percent) in Europe and Latin America with specific drug-overuse. A single patient can be diagnosed with more than one type of overuse. *p < 0.05

**Fig. 5:**
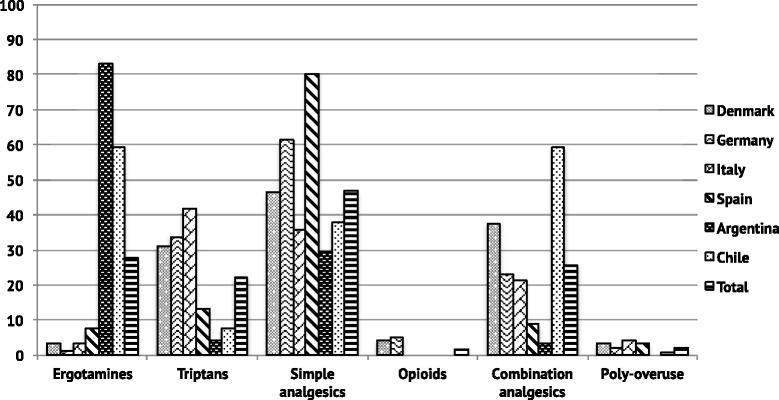
Type of overused analgesics. The bar chart compares the proportion of patients (in percent) from each headache clinic with specific drug-overuse. A single patient can be diagnosed with more than one type of overuse

While ergotamines were the most frequently overused analgesics among patients from LA (72.2 %), they were only overused by 3.7 % of the European patients with MOH (*p* < 0.001) (Fig. 4). In contrast, triptans were overused by 30.8 % of European patients with MOH and only by 5.6 % of LA patients (*p* < 0.001). The same pattern for ergotamines and triptans was seen when separating patients according to primary headache diagnosis (Table 3). Among the European patient groups, the Spanish had the lowest percentage of triptan overusers (13.0 %) and the highest percentage of ergotamine overusers (7.6 %) (Fig. 5). In contrast, Italy had the highest rate of triptan overusers among the European patients (41.9 %) (Fig. 5).

**Table 3:** MOH sub-diagnosis in relation to primary headache diagnosis

Primary headache diagnosis	Acute analgesics	Denmark	Germany	Italy	Spain	Argentina	Chile	Europe	Latin America	*P*-value	Total
N		38	81	97	43	91	86	259	177		436
Migraine	Ergotamines	5.3	1.2	4.1	2.3	81.3	62.8	3.1	72.3	<0.001*	31.2
	Triptans	55.3	37.0	47.4	16.3	4.4	8.1	40.2	6.2	<0.001*	26.4
	Simple analgesics	26.3	60.5	29.9	79.1	26.4	34.9	47.1	30.5	0.001*	40.4
	Opioids	5.3	6.2	0.0	0.0	0.0	0.0	2.7	0.0	0.045	1.6
	Combination-analgesics	18.4	21.0	21.6	4.7	3.3	62.8	18.1	32.2	0.001*	23.9
	Poly-overuse	5.3	2.5	3.1	4.7	0.0	1.2	3.5	0.6	0.54	2.3
N		33	2	1	15	7	2	51	9		60
TTH	Ergotamines	0.0	0.0	0.0	6.7	100.0	100.0	2.0	100.0	<0.001*	16.7
	Triptans	0.0	0.0	0.0	6.7	0.0	0.0	2.0	0.0	1.00	1.7
	Simple analgesics	81.8	50.0	100.0	86.7	42.9	0.0	82.4	33.3	0.005	75.0
	Opioids	3.0	0.0	0.0	0.0	0.0	0.0	2.0	0.0	1.00	1.7
	Combination-analgesics	36.4	100.0	0.0	13.3	0.0	50.0	31.4	11.1	0.42	28.3
	Poly-overuse	3.0	0.0	0.0	0.0	0.0	0.0	2.0	0.0	1.00	1.7
N		54	18	19	34	28	20	125	48		173
Migraine and TTH	Ergotamines	3.7	0.0	0.0	14.7	14.3	40.0	5.6	66.7	<0.001*	22.5
	Triptans	33.3	22.2	15.8	11.8	3.6	5.0	23.2	4.2	0.003*	17.9
	Simple analgesics	38.9	66.7	63.2	79.4	35.7	55.0	57.6	43.8	0.13	53.8
	Opioids	3.7	0.0	0.0	0.0	0.0	0.0	1.6	0.0	1.00	1.2
	Combination-analgesics	51.9	22.2	21.1	11.8	3.6	45.0	32.0	20.8	0.19	28.9
	Poly-overuse	1.9	0.0	10.5	2.9	0.0	0.0	3.2	0.0	0.58	2.3

Percentage of patients with specific MOH sub-diagnosis related to primary headache diagnosis. Number of each group are included in the table. Migraine includes forms with aura, without aura and chronic forms. TTH (Tension Type Headache) includes episodic and chronic forms. *p*-values correspond to comparison between Europe and Latin America. *p < 0.05

Simple analgesics were overused in all patient groups, corresponding to 46.9 % in the study population, and 15.4 % were daily users (Additional file 2: Table S2). In LA, 33.3 % of the patients overused simple analgesics (Fig. 4). However, significantly more patients from Europe (54.3 %) overused simple analgesics (*p* < 0.001), and noteworthy it was the case for 80.4 % of the Spanish group.

Combination-analgesics were often overused in Chile (59.3 %), Denmark (37.6 %), Germany (22.8 %) and Italy (21.4 %), while they were only overused by a minority of MOH patients in Spain (8.7 %) and Argentina (3.2 %) (Fig. 5). No significant difference in terms of overuse of combination-analgesics was observed between Europe and LA (*P* = 0.14), except among the group of patients with previous migraine (Table 3). In general, only a small proportion was diagnosed with an opioid-overuse (1.5 %) or poly-overuse (2.2 %). There was no significant difference in duration of medication overuse between Europe and LA (*P* = 0.13) (Additional file 2: Table S2). For the total population 59.8 % had a medication overuse for 1–5 years, and 23 % for more than 5 years.

The policy about whether a particular analgesic requires prescription is different among all the countries, as in the case with medication subsidization (summarized in Table 4).

**Table 4:** Medication pathways and subsidization

	Denmark	Germany	Italy	Spain	Argentina	Chile
OTC analgesics^a^	–	–	–	–	Ergotamines	Ergotamines^k^
	–	Triptans^d^		–	–	Triptans^l^
	Simple analgesics^b^	Simple analgesics^e^		Simple analgesics^g^	Simple analgesics	Simple analgesics
	–	–		–	–	–
	Combination-analgesics^c^	Combination-analgesics^f^		Combination-analgesics	–	Combination-analgesics^m^
Prescription needed	Ergotamines	Ergotamines	Ergotamines	Ergotamines^h^	–	–
	Triptans	Triptans^d^	Triptans	Triptans	Triptans	–
	Simple analgesics^b^	Simple analgesics^e^	Simple analgesics	–	–	–
	Opioids	–	Opioids	Opioids	Opioids	Opioids
	Combination-analgesics^c^	Combination-analgesics^f^	Combination-analgesics	–	Combination-analgesics	–
Analgesics with subsidization	Ergotamines	–	–	Ergotamines	–	–
	Triptans		Triptans	Triptans	Triptans^j^	
	–		–	Simple analgesics	Simple analgesics	
	Opioids		–	Opioids	Opioids	
	–		–	Combination-analgesics^i^	Combination-analgesics	
Analgesics without subsidization	–	Ergotamines	Ergotamines	–	Ergotamines	Ergotamines^k^
	–	Triptans	–	–	–	Triptans^l^
	Simple analgesics^b^	Simple analgesics	Simple analgesics	–	–	Simple analgesics
	–	Opioids	Opioids	–	–	Opioids
	Combination-analgesics^c^	Combination-analgesics	Combination-analgesics	Combination-analgesics^i^	–	Combination-analgesics^m^

^a^Over-the-counter analgesics

^b^Most simple analgesics are OTC. Prescription is only required for large packages or higher doses. Only prescribed simple analgesics are subsidized

^c^Combination-analgesics containing opioids, phenazone and ergotamine require prescription with a few exceptions. Only ergo-caffeine are subsidized

^d^Most triptans require prescription. Few triptans, e.g., Naratriptan, are OTC

^e^Most simple analgesics are OTC, except Ibuprofen 600 mg

^f^Combination-analgesics containing caffeine do not require prescription, while those containing opioids do

^g^Simple analgesics are OTC. However, prescription is used at least in 60 %

^h^Ergotamines require prescription. Nevertheless, many patients manage to acquire it without

^i^Some compounds are subsidized

^j^Triptans are only subsidized partially

^k^Ergotamines are inexpensive and easily available. All kind of ergotamins are combined with either caffeine, acetoaminophen or NSAIDS

^l^Triptans are expensive and available only at pharmacies

^m^Combination-analgesics are inexpensive. There are no combinations with codeine. Most common combination-analgesics contain caffeine, dipirone, chlorphenamine and ergotamine

Most patients in the study population only had a single MOH diagnosis (75.6 %), while 23 % had two MOH diagnoses and 1.5 % had three kind of medication overuse (Additional file 3: Table S3).

## Discussion

The findings in this study demonstrate a marked variability between Europe and LA regarding general and clinical characteristics of patients as well as the organization of healthcare systems, and headache-related healthcare utilization, medication-overuse and medications availability.

The patient groups showed significant differences in the distribution of primary headache diagnoses between Europe and LA countries. Considering the significantly higher level of education for LA patients and the fact that they have to pay out of their own pocket in order to receive the attention of a headache specialist, it is possible that the observed difference may be related to a different social class of subjects with higher income, more frequently single and tend to be younger. Of course cultural or real epidemiological differences cannot be ruled out.

The inhomogeneous distribution of primary headache diagnoses may theoretically have affected the proportions of the types of overused analgesics, since e.g., triptans and ergotamines are recommended for treatment for migraine only. However, when separating the groups according to primary headache diagnosis, we almost found the same results as in the whole group with a few exceptions as mentioned.

Only a minority of the patients reported pure TTH as primary headache. This could be due to less disability compared to patients with concomitant migraine, contributing to fewer referrals to headache centers. According to previously published Scandinavian studies concerning MOH, approximately 20–30 % percent suffered from pure TTH as primary diagnosis [17, 18], which is comparable to the findings in the Danish and Spanish group in this study.

Prior studies have characterized the use of healthcare in the general population [7, 10, 19], and our patients with MOH presented a multifold higher rate of access to headache specialists. This reflects that our study population was much more affected by headache, and emphasizes the unmet need for focused prevention and management strategies.

The fact that far more European patients had consultations with GPs and headache specialists compared with LA patients - while the latter more frequently access ER - may be due to different organization of the healthcare systems. Headache centers were non-existing in Chile (the first one was activated within the initiatives of the COMOESTAS Project 2008–2010) and only one headache center was available in Buenos Aires, a city with several millions of inhabitants. It must be noted that another possible reason for less GP and headache specialist contacts in LA could be less focus in society on headache as a disorder requiring therapy. We have observed a high frequency of EEG examinations in Germany in contrast to the existing guidelines [20] and practice in other countries. In general, EEG is not recommended for the diagnosis of headache but national practice may still vary.

In general, it seems that the healthcare system is not used in the appropriate manner, because a considerable proportion of MOH patients, both from Europe and LA, seek help at the ER, which does not seem as the optimal setting for managing a chronic condition. Furthermore, when considering the very high use of GP visits in some European countries, it could be important to clarify whether some European patients used the healthcare system at too high extent, thus contributing to an unnecessarily increased economic burden for society. In this case, a more focused effort to recognize and treat patients with MOH may contribute to fewer, but more relevant healthcare contacts.

It can be difficult to compare the use or ER accesses across different health care systems, since primary care systems and emergency systems may be fundamentally different. In this study there was free access in all countries. The findings concerning medication overuse in Europe are in line with results reported in previous studies from European countries [3, 7, 10]. In the present study, simple analgesics were overused in 80 % of the MOH patients from Spain, which is in agreement with the findings of a previous Spanish study, where simple analgesics also were the most overused drugs [3]. The results from the Danish patient group, showing highest preference for simple analgesics, followed by combination-analgesics, were supported by two previous Scandinavian studies [7, 10]. Also in the case of the ergotamine overuse in LA, our findings are reinforced by a recently published review that reports a high prevalence of ergotamine-overuse in LA, and a relatively high prevalence of triptan-overuse in Europe [21].

Ergotamines are highly potent drugs for treating migraine. In LA, ergotamines are sold as OTC analgesics at low prices in contrast to Europe where ergotamines require prescription. This may explain the widespread use in LA. In the eighties and nineties, overuse of ergotamines was a major problem in Europe, and a German study from 2002 reported that 13 % of the patients with MOH overused ergotamines [22]. The availability and, for some countries, the subsidization of triptans in Europe explain the marked decrease in ergotamine overuse and the associated increase in triptan overuse. The distribution policy of triptans, based on prescriptions, obviously did not succeed in deterring overuse. The ergotamine-triptan switch observed in Europe has also been reported in the United States [23], thus suggesting that replacement of ergotamines by triptans is likely to occur in the future in LA. It should be mentioned, that even though prescription was required for ergotamines in Spain, many patients managed to obtain ergotamines without. This could explain why patients from the Spanish group had the highest European proportion of ergotamine-overuse.

This study also reported other differences in the medication overuse pattern among the European countries. Italian patients tended to use more triptans than other European patients, which may be explained by the fact that triptans are the only analgesics with subsidization in Italy. Next to simple analgesics, triptans were the most often overused acute medication in Germany, even though there was no subsidization to triptans. Nevertheless, some triptans, e.g., naratriptan, are available as OTC analgesics. In Denmark, most simple analgesics and combination-analgesics are OTC analgesics, which probably made them the most often overused analgesics. The same phenomenon was observed in the German patient group. In Spain, the far most common overused analgesics were simple analgesics, which are available as OTC and subsidized.

In Argentina, simple analgesics are OTC analgesics, as in Denmark, Spain and Germany, and may also in this case contribute to overuse in almost one-third of the Argentinian patients. In Chile, combination-analgesics were overused almost as often as ergotamines. Both kinds of analgesics are sold as inexpensive OTC drugs.

Even though this study describes huge international variations in particular patterns of medication overuse, the underlying factors encouraging medication overuse seem to be the same in all countries: high availability and low prices. This is supported by previously published Asian studies, which reported a high prevalence of OTC analgesic overuse too [24–26]. Thereby, high availability and low costs of acute medication are global challenges for preventing MOH. When having the knowledge about which analgesics that are most commonly overused, specifically targeted clinical and political initiatives can be more easily initiated, e.g., smaller packages of analgesics and focused prevention campaigns spreading out information about the nature of MOH, thereby changing patients’ behavior.

### Methodological considerations

To our knowledge, no previous study has described and compared general demographic and clinical characteristics of MOH, along with the referral pathways and national painkillers distribution in several European and LA Countries. We did this multicenter, multinational setup in a large MOH population, which is a major strength of this study. The present study is unique as it describes patients accessing headache centers and specialized care, carefully classified by means of prospective diaries. The fact that the study was conducted on patients attending specialized center of course represents a selection bias making this study less applicable to patients in the primary sector and therefore prevents generalization of the results.

It is noteworthy that in the original study design MOH patients overusing barbiturates or pure opioid overuse were excluded, as well as patients with benzodiazepine overuse [13, 14]. This makes the study less applicable for specific groups of patients with high use of barbiturates, opioids and benzodiazepines. It should also be mentioned that patients with significant comorbid psychiatric illnesses were excluded. However, this study is applicable to patients with less complex psychiatric disorders and less severe MOH cases, representative for a significant part of the clinical MOH population in primary and specialized care. However, it may be difficult to compare use of emergency across health care systems due to different organizations of the link between the primary care sectors and the emergency systems. In some countries, there may be differences in how emergency rooms and emergency departments are organized. This study does not distinct between that.

The patients were included in 2008 and 2009. Some changes concerning acute medication overuse and healthcare utilization may have occurred since then. The present data remain however important as, to the best of our knowledge, no other study has provided relevant information on this topic in the subsequent years. It could be interesting to repeat the study for comparing with our results.

## Conclusion

MOH definitely represent a serious cross-continental issue, although demographic and clinical variability may exist, along with differences in referral patterns, use of healthcare resources and overuse of acute medications overuse in the 2 continents.

A clear message from our data is this: drugs are overused when they are easily accessible and at low price. A focused effort to recognize and treat patients with MOH may contribute to fewer, but more targeted healthcare contacts. Furthermore, clinical and political initiatives for awareness and prevention campaigns based on information of MOH can be better facilitated and targeted when having the knowledge about which types of analgesics that are more likely to become overused by headache sufferers in a particular part of the world.

## Additional files

Additional file 1: Table S1. Headache-related healthcare system utilization. Number of consultations per year are categorizes into 4 groups, and shown as percentage of the student population. ^1^Missing data on 3 patients from Germany and 5 patients from Spain, respectively. (PDF 55 kb) Additional file 2: Table S2. Medication overuse headache sub-diagnosis and duration of medication overuse. The table characterizes the medication overuse headache (MOH) sub-diagnosis. Results are shown as percent of patients with a specific MOH sub-diagnosis. Numbers in brackets presents the proportion of the patient population that used a specific analgesics 30 days/month. *p*-values correspond to comparison of MOH sub-diagnosis between Europe and Latin America. **p* < 0.05. ^1^A patient can have more than one MOH sub-diagnosis. ^2^Intake of combination of acute medication ≥ 15 days/month without overuse of a single drug. (PDF 67 kb) Additional file 3: Table S3. Multiple medication overuse headache sub-diagnoses and specific combination of medication overuse. The table characterizes the proportion of patients with multiple medication overuse headache (MOH) sub-diagnoses and the specific medication overuse profile. The data are shown as percentages. (PDF 66 kb)

## Abbreviations

CDH: chronic daily headache
COMOESTAS: Continuous Monitoring of Medication Overuse headache in Europe and Latin America
ER: emergency room
GP: general practitioner
LA: Latin America
MOH: medication overuse headache
OTC: over the counter drugs
TTH: tension type headache
