# Supplementary material for: Medication overuse headache in Europe and Latin America: general demographic and clinical characteristics, referral pathways and national distribution of painkillers in a descriptive, multinational, multicenter study
Source: J Headache Pain. 2016 Mar 8;17:20. doi: 10.1186/s10194-016-0612-2 (PMC4783306; doi:10.1186/s10194-016-0612-2)
Supplement: Additional file 1: Table S1. — Headache-related healthcare system utilization. Number of consultations per year are categorizes into 4 groups, and shown as percentage of the student population. 1Missing data on 3 patients from Germany and 5 patients from Spain, respectively. (PDF 55 kb) [file 10194_2016_612_MOESM1_ESM.pdf]

Supplementary Table 1 - Headache-related healthcare system utilization

		Denmark	Germany[1]	Italy	Spain[1]	Argentina	Chile	Total
	N	125	96	117	89	126	108	661
General practitioner consultations. Number/year	0	26.4	22.9	93.2	23.6	71.4	75.9	54.0
	1	27.2	10.4	6.0	9.0	10.3	14.8	13.3
	2–6	35.2	31.3	0.9	56.2	17.5	8.3	23.6
	> 6	11.2	35.4	0.0	11.2	0.8	0.9	9.1
Headache specialist consultations. Number/year	0	20.0	35.4	4.3	11.2	65.1	59.3	33.3
	1	35.2	20.8	91.5	46.1	24.6	31.5	41.9
	2–6	41.6	34.4	4.3	42.7	9.5	9.3	22.7
	> 6	3.2	9.4	0.0	0.0	0.8	0.0	2.1
Emergency department visits. Number/year	0	92.0	74.0	94.0	49.4	79.4	59.3	76.2
	1	2.4	9.4	4.3	25.8	7.1	18.5	10.4
	2–6	4.8	11.5	1.7	19.1	11.9	20.4	11.9
	> 6	0.8	5.2	0.0	5.6	1.6	1.9	2.3

Number of consultations per year are categorizes into 4 groups, and shown as percentage of the student population. [1]Missing data on 3 patients from Germany and 5 patients from Spain, respectively.
