# Supplementary material for: Medication overuse headache in Europe and Latin America: general demographic and clinical characteristics, referral pathways and national distribution of painkillers in a descriptive, multinational, multicenter study
Source: J Headache Pain. 2016 Mar 8;17:20. doi: 10.1186/s10194-016-0612-2 (PMC4783306; doi:10.1186/s10194-016-0612-2)
Supplement: Additional file 2: Table S2. — Medication overuse headache sub-diagnosis and duration of medication overuse. The table characterizes the medication overuse headache (MOH) sub-diagnosis. Results are shown as percent of patients with a specific MOH sub-diagnosis. Numbers in brackets presents the proportion of the patient population that used a specific analgesics 30 days/month. p-values correspond to comparison of MOH sub-diagnosis between Europe and Latin America. *p < 0.05. 1A patient can have more than one MOH sub-diagnosis. 2Intake of combination of acute medication ≥ 15 days/month without overuse of a single drug. (PDF 67 kb) [file 10194_2016_612_MOESM2_ESM.pdf]

Supplementary table 2 - Medication overuse headache sub-diagnosis and duration of medication overuse

		Denmark	Germany	Italy	Spain	Argentina	Chile	Europe	Latin America	p-value	Total
	N	125	101	117	92	126	108	435	234		669
[1]MOH diagnosis	Ergotamines	3.2	1.0	3.4	7.6	83.3	59.3	3.7	72.2	<0.001*	27.7
		(0.8)	(1.0)	(0.9)	(2.2)	(31.7)	(14.8)	(1.2)	(23.9)		(9.1)
	Triptans	31.2	33.7	41.9	13.0	4.0	7.4	30.8	5.6	< 0.001*	22.0
		(3.2)	(6.9)	(6.8)	(0.0)	(0.0)	(0.9)	(4.1)	(0.4)		(2.8)
	Simple	46.4	61.4	35.9	80.4	29.4	38.0	54.3	33.3	< 0.001*	46.9
	analgesics	(25.6)	(26.7)	(20.5)	(15.2)	(10.3)	(8.3)	(18.2)	(10.3)		(15.4)
	Opioids	4.0	5.0	0.0	0.0	0.0	0.0	2.3	0.0	0.018*	1.5
		(0.8)	(3.0)	(-)	(-)	(-)	(-)	(0.9)	(-)		(0.6)
	Combination	37.6	22.8	21.4	8.7	3.2	59.3	23.7	29.1	0.14	25.6
	analgesics	(19.4)	(7.9)	(7.7)	(3.3)	(0.8)	(11.1)	(10.2)	(5.6)		(8.5)
	[2]Poly-overuse	3.2	2.0	4.3	3.3	0.0	0.9	3.2	0.4	0.025*	2.2
Duration of overuse in years	< 1	13.6	21.8	11.1	16.3	19.0	22.2	15.4	20.5		17.2
	1 - 5	67.2	54.5	58.1	57.6	61.9	57.4	59.8	59.8	0.13	59.8
	> 5	19.2	22.4	30.8	26.1	19.0	20.4	24.8	19.7		23.0

The table characterizes the medication overuse headache (MOH) sub-diagnosis.
Results are shown as percent of patients with a specific MOH sub-diagnosis. Numbers in brackets presents the proportion of the patient population that used a specific analgesics 30days/month.
p-values correspond to comparison of MOH sub-diagnosis between Europe and Latin America. *$p < 0.05$.
[1]A patient can have more than one MOH sub-diagnosis. [2]Intake of a combination of acute medication ≥ 15 days/month without overuse of a single drug.
