# Supplementary material for: Medication overuse headache in Europe and Latin America: general demographic and clinical characteristics, referral pathways and national distribution of painkillers in a descriptive, multinational, multicenter study
Source: J Headache Pain. 2016 Mar 8;17:20. doi: 10.1186/s10194-016-0612-2 (PMC4783306; doi:10.1186/s10194-016-0612-2)
Supplement: Additional file 3: Table S3. — Multiple medication overuse headache sub-diagnoses and specific combination of medication overuse. The table characterizes the proportion of patients with multiple medication overuse headache (MOH) sub-diagnoses and the specific medication overuse profile. The data are shown as percentages. (PDF 66 kb) [file 10194_2016_612_MOESM3_ESM.pdf]

Supplementary Table 3 - Multiple medication overuse headache sub-diagnoses and specific combination of medication overuse

**Combination of MOH diagnoses**	**Denmark**	**Germany**	**Italy**	**Spain**	**Argentina**	**Chile**	**Total**
N	125	101	117	92	126	108	669
Single MOH type	74.4	78.2	93.2	87.0	81.7	38.9	75.6
Two MOH types	24.8	17.8	6.8	13.0	17.5	57.4	22.9
Three MOH types	0.8	4.0	0.0	0.0	0.8	3.7	1.5
Triptans and ergotamines	0.0	0.0	0.0	2.2	1.6	0.9	0.7
Triptans and simple analgesics	5.6	9.9	2.6	4.3	0.8	0.9	3.9
Triptans and combination-drugs	4.0	1.0	1.7	0.0	0.0	0.9	1.3
Triptans and poly-overuse	1.6	0.0	0.0	0.0	0.0	0.0	0.3
Ergotamines and simple analgesics	0.8	0.0	0.9	3.3	14.3	1.9	3.7
Ergotamines and combination-drugs	0.8	0.0	0.0	1.1	0.8	45.4	7.8
Simple analgesics and opioids	2.4	2.0	0.0	0.0	0.0	0.0	0.7
Simple analgesics and combination-drugs	8.8	3.0	1.7	2.2	0.0	6.5	3.7
Simple analgesics and poly-overuse	0.8	0.0	0.0	0.0	0.0	0.0	0.1
Opioids and combination-drugs	0.0	1.0	0.0	0.0	0.0	0.0	0.1
Combination-drugs and poly-overuse	0.0	1.0	0.0	0.0	0.0	0.0	0.3
Ergotamines, simple analgesics and combination-drugs	0.0	0.0	0.0	0.0	0.0	2.8	0.4
Triptans, simple analgesics and combination-drugs	0.8	1.0	0.0	0.0	0.0	0.9	0.4
Simple analgesics, opioids and combination-drugs	0.0	1,0	0.0	0.0	0.0	0.0	0.1
Triptans, simple analgesics and opioids	0.0	1,0	0.0	0.0	0.0	0.0	0.1
Ergotamines, triptans and simple analgesics	0.0	1,0	0.0	0.0	0.8	0.0	0.3

The table characterizes the proportion of patients with multiple medication overuse headache (MOH) sub-diagnoses and the specific medication overuse profile. The data are shown as percentages.
